# The role of postoperative sucralfate in adults following tonsillectomy and sleep surgery: a systematic review

**DOI:** 10.1007/s00405-025-09311-1

**Published:** 2025-03-28

**Authors:** Lauren R. McCray, Erin E. Briggs, Jaimin J. Patel, Shaun A. Nguyen, Noah Parker

**Affiliations:** 1 Department of Otolaryngology-Head and Neck Surgery, Medical University of South Carolina, 135 Rutledge Avenue, Room 1133, MSC 550, Charleston, SC 29425-5500 USA; 2 Baylor College of Medicine, Houston, TX USA; 3 Thomas Jefferson University School of Medicine, Philadelphia, PA USA; 4 Indiana University School of Medicine, Indianapolis, IN USA

**Keywords:** Quality of life, Oropharynx, Tonsillectomy, Systematic review, Sucralfate

## Abstract

**Purpose:**

To investigate the efficacy of topical sucralfate on postoperative recovery following oropharyngeal surgery in adults using pain scales, analgesic use, and various self-reported measures.

**Methods:**

CINAHL, Cochrane Library, PubMed, and SCOPUS databases were searched from inception through July 3, 2024. Randomized controlled trials related to topical sucralfate following oropharyngeal surgery in patients at least 18 years old were included. Study protocols for clinical trials, abstracts, and non-English language articles were excluded. Two authors extracted data, and disagreements were resolved with a third party if needed. Risk of bias was assessed according to Risk of Bias 2 (RoB 2) tool. Results of included studies and a narrative summary of our findings are presented through descriptive statistics (frequency (%) for categorical variables and mean (range) for continuous variables).

**Results:**

Four studies (*n* = 185) pertaining to topical sucralfate and post-operative outcomes in an adult population were included. The sucralfate group had a mean age of 40.08 vs. 37.50 for the control group. The sucralfate group had a significantly higher reduction in pain scores than the control group. The sucralfate group also had statistically significant improvements in otalgia, strength, diet tolerance, and reduction in analgesic use compared to the control group in two of the four studies.

**Conclusions:**

Oropharyngeal surgery is commonly performed in adults despite having a morbid recovery process. The literature shows promising results with the use of sucralfate in the reduction of post-operative pain in adults; however, further investigation is warranted given the limited scope of the literature.

## Introduction

Oropharyngeal surgery is commonly performed for recurrent tonsillitis and obstructive sleep apnea (OSA) in adults. In the United States, 297,000 of the 737,000 tonsillectomies done in 2006 were on patients 15 years or older [1]. In addition, uvulopalatopharyngoplasty (UPPP) is one of the most common surgical treatments for OSA in adults. Although tonsillectomies and UPPP are generally safe, with 30-day mortality rates of 0.03% and 0.09%, respectively, they can have high morbidity [2–3]. Throat pain is a common post-operative complaint, which can negatively impact quality of life and patient comfort, contributing to delayed recovery. Current pain management for oropharyngeal surgeries includes steroids, acetaminophen with codeine, ibuprofen, and scheduled opioid analgesics [4]. The scheduled use of opioids is not without risk as it can induce life-threatening respiratory depression. The current paradigm in postoperative treatment may not be effective at promoting pain management and mucosal recovery, so it is important to investigate alternative treatments [5, 6].

Sucralfate is FDA-approved for duodenal ulcers, and it acts by creating a protective physical barrier, enhancing tissue growth through regeneration and repair, as well as inhibiting bacterial activity [7]. The first use of topical sucralfate was reported in 1991 for stomal or perineal skin ulceration after other agents had failed. It was successful in 14 out of 15 patients with no toxic or systemic effects [8]. Topical sucralfate also has clinical benefits for various mucocutaneous conditions such as burn injuries and post-radiotherapy reactions [7]. In addition, sucralfate has been used post-operatively for pediatric tonsillectomies, with improvements in pain reduction, bleeding rates, diet tolerance, and need for additional analgesics [9–11]. Given its efficacy in promoting mucosal healing, sucralfate could aid in the recovery from oropharyngeal surgery in adults. However, there is a paucity of data on the use of sucralfate in this setting.

The current literature on the use of sucralfate for adult patients undergoing oropharyngeal surgery is limited. This systematic review aims to investigate the efficacy of sucralfate on various post-operative measures such as throat pain, analgesic use, mucosal coverage and bleeding following oropharyngeal surgeries in adults. In doing so, we aim to highlight the need for further research on sucralfate in this population.

## Materials and methods

This study was conducted according to the Preferred Reporting Items for Systematic Reviews and Meta-Analyses (‘PRISMA’) guidelines [12]. A preliminary search of PubMed and the Cochrane Database of Systematic Reviews was conducted, and no current systematic reviews on the topic were identified.

### Identify research question

This review sought to investigate the effect of topical sucralfate on various clinically relevant post-operative measures such as pain, analgesic use, and bleeding following oropharyngeal surgery in adults. Furthermore, we wanted to highlight the clinical importance of the limitations in the current literature.

### Identify relevant literature

A systematic search was conducted with PubMed (US National Library of Medicine, National Institutes of Health), SCOPUS (Elsevier), CINAHL (EBSC), and Cochrane databases on 3 July 2024 by authors LRM and EEB, using keywords “sucralfate” AND (“tonsillectomy” OR “uvulopalatopharyngoplasty” OR “UPPP” OR “expansion pharyngoplasty” OR “sleep surgery” OR “sleep procedure”).

All articles from the search were exported into Covidence (Veritas Health Innovation Ltd., Melbourne, Australia), the review management software, for screening. All identified citations were collated and uploaded in EndNote X20/2021 (Clarivate Analytics, Philadelphia, PA, USA).

### Study selection

This study aimed to identify all published reports relevant to the use of topical sucralfate following oropharyngeal surgery. The population of interest was adults (*≥* 18 years). This scoping review considered randomized controlled trials, non-randomized controlled trials, prospective and retrospective cohort studies, prospective and retrospective chart reviews, case–control studies, and case series studies. Review articles were assessed but not included in the reporting of quantitative data to avoid redundancy. Other exclusion criteria were patients < 18 years old, study protocols for clinical trials, abstracts, non-English language articles, and incomplete or inaccessible articles.

#### Search process

The initial search yielded 220 studies with the removal of 27 duplicates. The remaining 193 titles and abstracts were screened by two independent reviewers (EEB and LRM) for assessment against the exclusion criteria, and conflicts were resolved through discussion. There were 7 full-text studies assessed for eligibility, with 1 being excluded at the full-text stage for wrong population and 2 being excluded for having a pediatric population. Four full-text screenings were completed independently by the same reviewers (EEB and LRM) and any disagreements were resolved through discussion. The results of the study inclusion process are presented in a PRISMA flow diagram.

### Charting the data

#### Data extraction

Data extraction from the four studies was performed independently by two authors (LRM and EEB), and disagreements were resolved through the third author (JJP). The data extracted from reports included basic study information such as author, year of publication, study design, type of procedure performed, and patient demographics, including age and gender. Post-operative measures such as pain scores, type of analgesic, frequency of analgesic use, mucosal coverage and bleeding were also extracted.

#### Level of evidence and risk of bias

All the included reports were critically appraised to assess the level of evidence using the Oxford Center for Evidence-Based Medicine criteria [13]. The risk of bias was assessed according to the Cochrane Handbook for Systematic Reviews of Interventions (version 6.0.22) [14]. The level of evidence and the risk of bias were assessed by author EEB and verified for accuracy by author LRM (Fig. 2). The Risk of Bias 2 (RoB 2) tool was used to evaluate the randomized study designs [15]. Risk-of-bias items included the following: bias due to confounding, bias in selection of participants of the study, bias in classification of interventions, bias due to deviations from intended interventions, bias due to missing data, bias in measurement of outcomes, and bias in selection of the reported results. The risk of bias for each category is graded as low risk, high risk, or unclear risk. Articles were not excluded based on quality since a meta-analysis was not performed.

### Collating, summarizing and reporting results

Relevant information from the included studies was narratively synthesized following guidance from the Cochrane Handbook for summarizing findings when meta-analysis was not feasible. Results of included studies and a narrative summary of our findings are presented through descriptive statistics (frequency (%) for categorical variables and mean (range) for continuous variables with 95% confidence intervals (CI)). Qualitative data were synthesized in a narrative format. Implications of the analysis are then discussed regarding the role of sucralfate in postoperative outcomes in adults following oropharyngeal surgery.

## Results

### Study characteristics

**Fig. 1:**
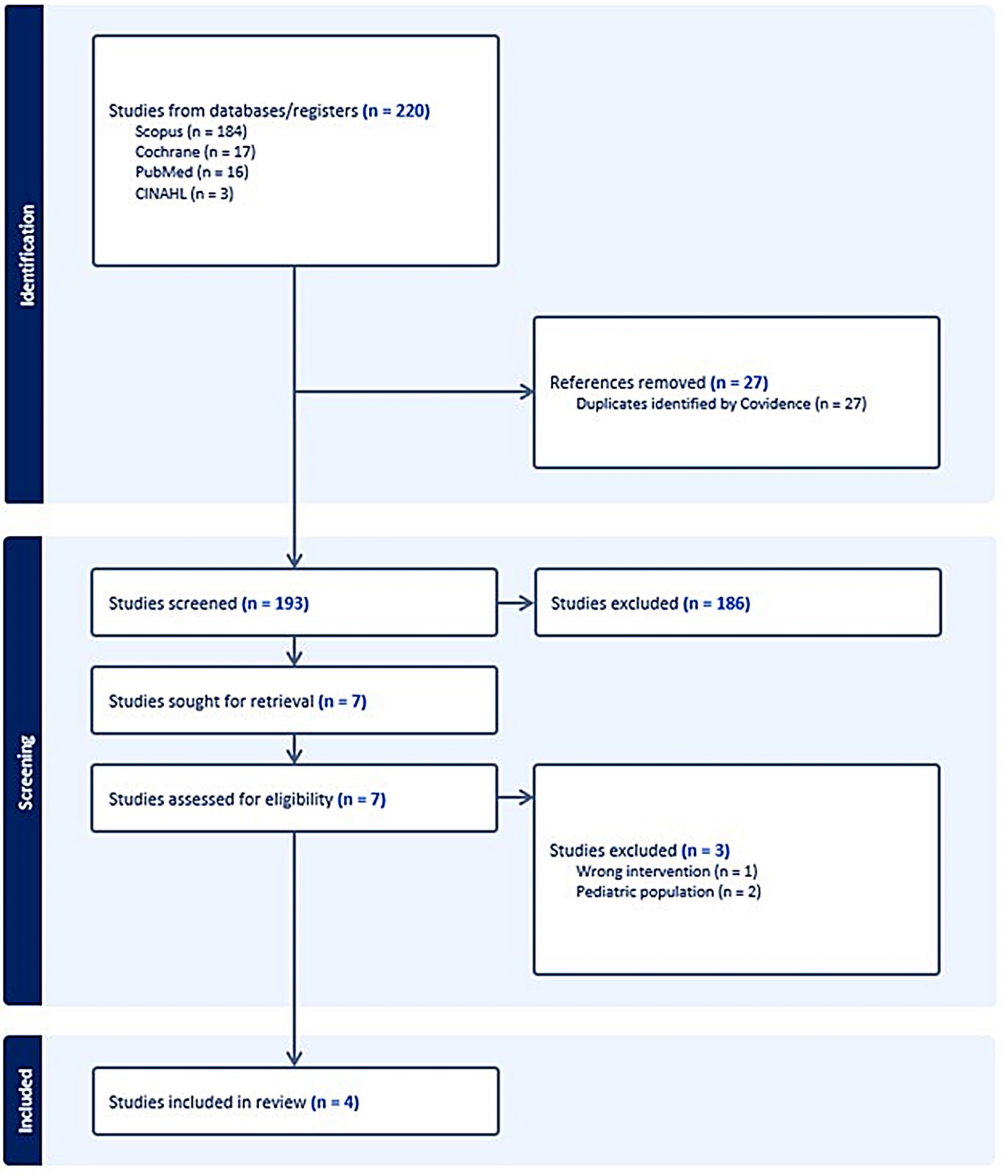
Preferred reporting items for systematic reviews and meta-analyses (‘PRISMA’) diagram

The four studies in our review were randomized control trials published between 1992 and 2006 [16–19]. A PRISMA diagram outlining our search is provided in Fig. 1. Descriptions of the individual studies and selected patient characteristics are shown in Table 1.

**Table 1:** Demographics of included studies

Study	Oxford Level of Evidence	Number of Patients	Procedure	Outcomes
Candan et al. [16]	2	43	Tonsillectomy	Pain, otalgia, trismus, reepithelization, wound edema
Freeman and Markwell [17]	2	34	Tonsillectomy	Pain, mucosal coverage, bleeding, diet
Kyrmizakis et al. [18]	2	28	Laser-assisted uvulopalatoplasty (LAUP)	Pain, analgesic use, bleeding, diet
Zodpe et al. [19]	2	80	Uvulopalatopharyngoplasty (UPPP)	Pain, analgesic use, mucosal coverage, bleeding

### Patient attributes

The studies include adult patients undergoing oropharyngeal surgery. There were 185 patients: 90 received topical sucralfate, 84 received a placebo, and 11 received neither sucralfate nor placebo. The sucralfate group was 69.7% male (95% CI: 43.5-90.3%) and the placebo group was 72.0% male (95% CI: 40.4-94.6%). The average age of the combined placebo and control group was 37.5 (range: 27–64 years), comparable to the sucralfate group with an average age of 40.1 (range: 28–65 years). 77 patients underwent tonsillectomy, 28 underwent laser-assisted uvulopalatoplasty (LAUP), and 80 underwent uvulopalatoplasty (UPPP).

### Quality assessment

**Fig. 2:**
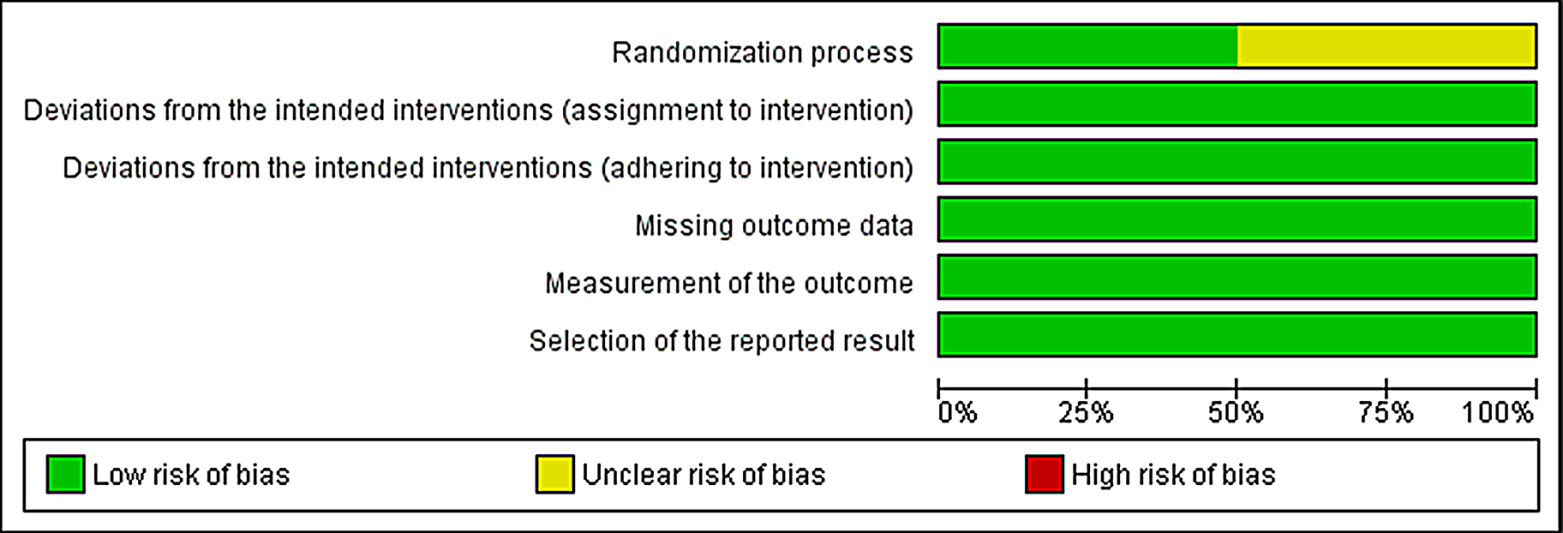
Risk of bias graph: review authors’ judgments about each risk of bias item presented as percentages across all included studies

Based on Oxford Level of Evidence criteria, all four studies are level 2 evidence (Table 1). Risk of bias was assessed for each study; two studies had an unclear risk of bias arising from the randomization process (Fig. 2) [16–17].

### Outcomes

#### Candan et al. [16]

Patients (*n* = 43) who underwent tonsillectomy were divided into three groups: two groups were given a 50 milliliter (mL) irrigation solution containing either one gram of sucralfate (*n* = 20) or one gram of lactose (*n* = 12) to take four times daily, and one group did not receive any medication (*n* = 12). Patients reported throat pain, otalgia, and trismus twice a day for 5 days. Trained clinical personnel estimated the percentage of reepithelization and wound edema covering the tonsillar fossa on post-operative day 10 through direct visualization. All outcomes were rated on a 0–4 scale with 0 corresponding to none, 1 to mild, 2 to moderate, 3 to severe, and 4 to very severe. Outcomes were reported as the number of patients who had average scores of 0–1 or 2 and up. There was not a significant difference in throat pain relief between the sucralfate and other two groups until after the morning of the second postoperative day. There was no significant difference in otalgia and trismus outcomes during the first five postoperative days, but there was a significant improvement in epithelization and wound edema on post-operative day 10 in the sucralfate group compared to the placebo and control groups.

#### Freeman and Markwell [17]

Patients (*n* = 34) who underwent tonsillectomy were given a 60 mL irrigation solution containing either one gram of sucralfate (*n* = 16) or one gram of lactose (*n* = 18) to take four times daily for ten days. Patients reported throat pain, otalgia, trismus, milliliters of pain medication, type of diet and percent of diet eaten, percent of strength return, bleeding, and temperature twice a day for 10 days. Trained clinical personnel weighed patients and estimated the percentage of mucosa covering the tonsillar fossa on post-operative day 10. Similar to *Candan et al.*, throat pain, otalgia, and trismus were rated on a 0 to 4 scale with 0 corresponding to none and 4 to very severe. There was a reduction in throat pain, otalgia, and trismus in the sucralfate group compared to the placebo, with a consistently significant result for throat pain and a significant difference emerging after day 1 for trismus and day 3 for otalgia. The milliliters of acetaminophen and codeine required were significantly less in the sucralfate group only for the first 1.5 days after surgery. There were 10 patients who reported gaining half of their strength in the sucralfate group on post-operative day 10 compared to 8 patients in the control group, which was a significant difference. As shown in Table 2, the average number of days at which patients were able to eat half of their normal diet was 6 for the sucralfate group and 8 for the control group, which was also significantly different. There was no significant difference between sucralfate and placebo regarding type of diet, weight change, number of bleeding episodes, temperature, and percent mucosal coverage.

**Table 2:** Diet outcomes from the freeman and Kyrmizakis studies, respectively

Patient Group	Average number of days at which patients were able to eat 50% of their normal diet	Average number of days at which patients were able to eat 80% of their normal diet
Sucralfate	6	8
Control	8	10

#### Kyrmizakis et al. [18]

Patients (*n* = 28) who underwent LAUP received an oral suspension containing sucralfate (1 g/5 mL) every 6 h for 15 days (*n* = 14) or a placebo with the same dosing (*n* = 14). Patients reported throat pain, the number of paracetamol tablets received, and the presence of upper abdominal upset every day for 5 days. The time patients reached 80% of their normal diet quantity and the presence of bleeding was evaluated on postoperative day 15. Pain was measured on a 3-point scale, with 0 representing no pain and 3 representing maximal pain. As shown in Table 3, there was a significant difference between the sucralfate and placebo groups in terms of average pain scores, frequency of analgesic use, and number of patients with severe otalgia, all of which were assessed on postoperative day 5. As shown in Table 3, the average number of days at which patients were able to eat 80% of their normal diet was 8 for the sucralfate group and 10 for the placebo group, which was a significant difference. There was also a significant difference in the number of patients reporting abdominal discomfort, with one in the sucralfate group compared to six in the placebo group on postoperative day 5. There was no difference in episodes of postoperative bleeding.

**Table 3:** Selected outcomes from the Kyrmizakis study

Patient Group	Average Pain Score (0–3) on POD 5	Frequency of Analgesic Use on POD 5	Number of Patients with Severe Otalgia on POD 5
Sucralfate (*n* = 14)	1.14	2.14	5
Control (*n* = 14)	2.07	4.43	12

#### Zodpe et al. [19]

Patients (*n* = 80) who underwent UPPP received a 60 mL oral suspension containing one gram of sucralfate (*n* = 40) or a placebo (*n* = 40) every six hours for six days. Patients reported throat pain and otalgia on a 7-point scale from post-op day 0 to 6, with 0 representing no pain and 7 representing maximal pain. The number of ibuprofen tablets, degree of strength, percentage of mucosal coverage, and postoperative bleeding were also recorded daily for 6 days. Degree of strength was reported on 3-point scale, with 1 meaning that the patient felt well and 3 meaning the patient felt weakness. Mucosal coverage was graded on a 5-point scale from 0 to 4, with 0 corresponding to complete healing and 4 corresponding to 76–100% bare mucosa. As shown in Table 4, there was a significant reduction in throat pain between the control and sucralfate groups from post-op days 3–6. There was a significant reduction in otalgia from postoperative days 0–6. There was a significant increase in strength from postoperative days 4–6. There was a significant reduction in analgesic requirement from post-operative days 1–3. There was no significant difference between sucralfate and control groups in terms of late postoperative bleeding.

**Table 4:** Selected outcomes from the Zodpe et al. [19] study

	Throat Pain		Analgesic Requirement		Otalgia	
POD	c	s	c	s	c	s
0	6.0	5.8	4.0	4.0	4.3*	0.5*
1	5.8	5.3	4.0*	3.0*	4.0*	0.3*
2	5.3	4.8	4.0*	3.0*	3.8*	0*
3	4.5*	3.3*	2.0*	2.0*	3.3*	0*
4	4.3*	3.0*	2.8	2.0	3.0*	0*
5	4.0*	2.0*	1.8	2.0	2.8*	0*

Abbreviations: POD = post-operative day, c = control, s = sucralfate. Asterisks indicate a statistically significant difference (*p* < 0.05) between the control and sucralfate groups

## Discussion

The aim of this systematic review is to assess the efficacy of sucralfate on reducing post-operative morbidity and facilitating recovery in adults undergoing oropharyngeal surgery. Various pain management strategies for tonsillectomy and UPPP patients have been investigated, such as pre- and intra-operative paracetamol, dexamethasone, and gabapentinoids [20]. A recent systematic review and meta-analysis on pain management strategies for tonsillectomy patients found that NSAIDs or intraoperative steroids offered significant pain relief compared to alternative medications or placebo, respectively, in the first 24 h and the third day following surgery [21]. However, in a recent cohort study of patient-reported outcomes following tonsillectomy, the most common complaint among dissatisfied patients was analgesics not being sufficiently helpful. Adult patients who received opioids were more likely to be satisfied with pain management but also experienced more side effects [5].

Given the inadequacy of current pain management strategies following oropharyngeal surgery, sucralfate may benefit patients as an additional therapeutic agent in the postoperative period. Among the two studies that involved tonsillectomies, both reported significant relief from throat pain with sucralfate. One study reported significant improvement in epithelization and reduction in wound edema, while another reported significant reduction in otalgia, trismus, and use of acetaminophen and codeine in the first 1.5 postoperative days as well as significant decrease in time needed for return to normal diet and recovery of strength. Among the studies that reported on LAUP and UPPP, both reported significant decreases in throat pain, otalgia, and need for analgesics among the sucralfate group; one study reported significant improvement in time to return to normal diet and decreased episodes of abdominal discomfort, while another reported significant improvement in return to normal strength.

Two randomized controlled trials investigated the use of sucralfate in both pediatric and adult tonsillectomy patients; however, they were not included in our review because they did not stratify outcomes based on age. Harshvardhan et al. (2022) studied 64 patients ranging from 6 to 50 years old and found significant differences in throat pain, otalgia, and trismus [22]. Ozcan et al. (1998) studied 89 patients ranging from 12 to 46 years old and found significant differences in throat pain and analgesic requirement [23]. Although neither of these studies were included in our review, both of them found promising results with sucralfate, especially for throat pain.

Despite advances in surgical techniques and pain management strategies, oropharyngeal surgery is highly morbid. The 30-day overall complication rate for tonsillectomies in adults has been reported as 1.2%, with the most common complications being due to infections (58%), and 16% of overall complications comprised superficial site infections [2]. The 30-day overall medical or surgical complication rate for UPPP was 1.4%, with 0.7% of patients experiencing surgical complications including superficial surgical site infection, deep surgical site infection, wound dehiscence, or bleeding requiring blood transfusion [3]. Given sucralfate’s antibacterial properties, it may help lower post-operative infection rates and has already been demonstrated as efficacious in preventing oral mucositis among patients undergoing cancer therapy [24]. In addition, since one study reported benefits of sucralfate for re-epithelization, this may translate to improved pain control as the underlying pharyngeal muscles are less exposed. Thus, sucralfate may have benefits through its multi-faceted actions.

There are limitations to our review. The heterogeneity between the studies makes it difficult to compare the results; for example, there were differences in the frequency and length of post-operative follow-up ranging from once a day for five days to twice a day for ten days. In addition, not all studies investigated the same post-operative outcomes, and even with overlapping outcomes, the maximum score on patient-reported scales ranged from 3 to 7. Another limiting factor amongst the studies were the small cohorts. They ranged from 28 to 80 patients, which limits the statistical power when comparing sucralfate to standard of care. Furthermore, all studies were published several decades ago; the most recent study was conducted in 2007. We believe this may be due to difficulties prescribing sucralfate for oropharyngeal surgery. The liquid suspension, which is more expensive than tablets, is oftentimes not covered by insurance for this indication, which makes it difficult to access, not only for conducting research, but also for patient care.

## Conclusion

Topical sucralfate has demonstrated benefits in the post-operative period for adults undergoing tonsillectomies and UPPPs. Otolaryngologists performing these procedures should consider using sucralfate given its potential benefits for throat pain, otalgia, analgesic use, and re-epithelization. In addition to producing more data to guide future clinical management, more widespread use of sucralfate could enhance the wellbeing of these patients. Therefore, we recommend further investigation and use of sucralfate in clinical practice. More extensive insurance coverage for topical sucralfate in patients undergoing oropharyngeal surgery could increase accessibility to this medication for clinical and research purposes.
